# 4-Bromo-2*H*-1,3-oxazine-2,6(3*H*)-dione

**DOI:** 10.1107/S1600536809034631

**Published:** 2009-09-05

**Authors:** Damon Parrish, Parcharee Tivitmahaisoon, Gretchen M. Rehberg, Margaret E. Kastner

**Affiliations:** a Department of Chemistry, Bucknell University, Lewisburg, PA 17837, USA

## Abstract

The title compound, C_4_H_2_BrNO_3_, is one of a series of three substituted oxauracils prepared as precursors in the preparation of 1-aza-1,3-butadienes. Although each structure has identical potential for N—H⋯O inter­molecular hydrogen bonds, each forms a distinctive inter­molecular network. In the title compound, there are two independent mol­ecules in the asymmetric unit, with a non-crystallographic twofold screw-like relationship between them. The two indpendent mol­ecules are linked by an inter­molecular N—H⋯O hydrogen bond. In the crystal structure, this hydrogen-bonded pair is linked to translationally related mol­ecules through further inter­molecular N—H⋯O hydrogen bonds, forming one-dimensional chains along [100]. The crystal structure also has short Br⋯O=C inter­molecular contacts with distances of 2.843 (4) and 2.852 (4) Å.

## Related literature

For the crystal structures of related oxauracils, see: Parrish, Leuschner *et al.* (2009 ▶); Parrish, Glass *et al.* (2009 ▶); Copley *et al.* (2005 ▶); Yathirajan *et al.* (2007 ▶). For synthetic details, see: Rehberg & Glass (1995 ▶); Warren *et al.* (1975 ▶). For a description of the Cambridge structural Database, see: Allen (2002 ▶).
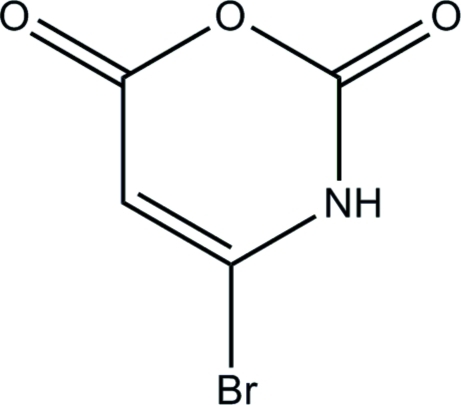


## Experimental

### 

#### Crystal data

C_4_H_2_BrNO_3_
*M* *_r_* = 191.98
Orthorhombic, 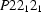

*a* = 7.8913 (12) Å
*b* = 11.8481 (16) Å
*c* = 12.264 (2) Å
*V* = 1146.6 (3) Å^3^
*Z* = 8
Mo *K*α radiation
μ = 7.09 mm^−1^
*T* = 293 K
0.45 × 0.20 × 0.10 mm

#### Data collection

Siemens R3m/V diffractometer
Absorption correction: ψ scan (*SADABS*; Bruker, 2000 ▶) *T*_min_ = 0.246, *T*_max_ = 0.492
2649 measured reflections
2649 independent reflections
1975 reflections with *I* > 2σ(*I*)
3 standard reflections every 50 reflections intensity decay: none

#### Refinement

*R*[*F* ^2^ > 2σ(*F* ^2^)] = 0.038
*wR*(*F* ^2^) = 0.092
*S* = 0.95
2649 reflections
163 parameters
H-atom parameters constrained
Δρ_max_ = 0.85 e Å^−3^
Δρ_min_ = −0.53 e Å^−3^
Absolute structure: Flack (1983 ▶), 1123 Friedel pairs
Flack parameter: 0.000 (17)

### 

Data collection: *XSCANS* (Bruker, 2000 ▶); cell refinement: *XSCANS*; data reduction: *SHELXTL* (Sheldrick, 2008 ▶); program(s) used to solve structure: *SHELXS97* (Sheldrick, 2008 ▶); program(s) used to refine structure: *SHELXL97* (Sheldrick, 2008 ▶); molecular graphics: *SHELXTL*; software used to prepare material for publication: *SHELXTL*.

## Supplementary Material

Crystal structure: contains datablocks global, I. DOI: 10.1107/S1600536809034631/lh2885sup1.cif

Structure factors: contains datablocks I. DOI: 10.1107/S1600536809034631/lh2885Isup2.hkl

Additional supplementary materials:  crystallographic information; 3D view; checkCIF report

## Figures and Tables

**Table 1:** Hydrogen-bond geometry (Å, °)

*D*—H⋯*A*	*D*—H	H⋯*A*	*D*⋯*A*	*D*—H⋯*A*
N3—H3⋯O2*A*	0.86	1.99	2.841 (6)	171
N3*A*—H3*A*⋯O2^i^	0.86	2.05	2.903 (6)	169

Symmetry code: (i) 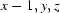.

**Fig. 1.**
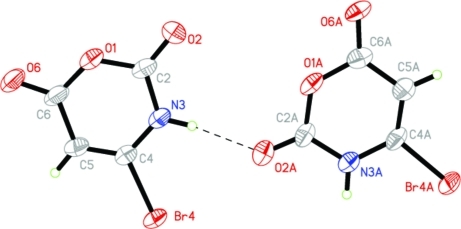
The molecular structure of the title compound, with atom labels and 50% probability displacement ellipsoids for non-H atoms. The dashed line indicates a hydrogen bond.

**Fig. 2.**
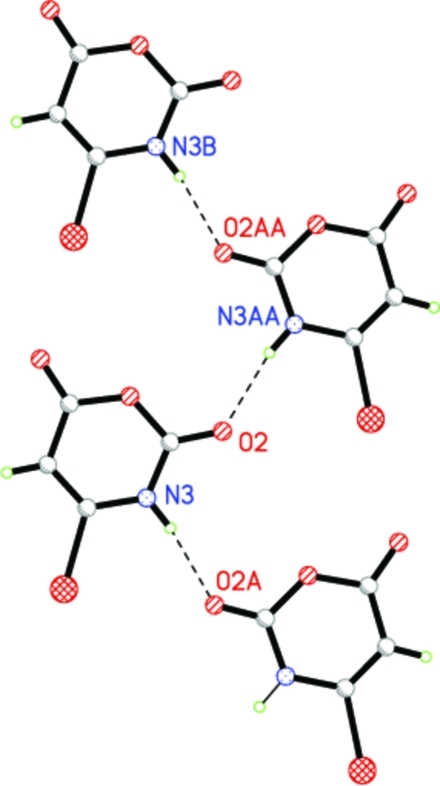
The two indetendent molecules in the asymmetric unit plus a pair related by translation along the a axis (O2A is identical to O2AA by translation, as are N3 and N3B). The psuedo-2-fold screw runs approximately through O2 and O2A.

**Fig. 3.**
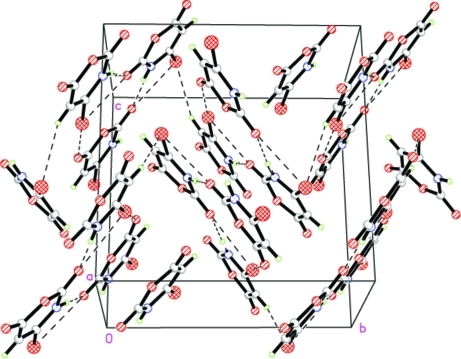
Packing diagram of the title compound viewed approximately along [100]. Dashed lines indicate hydrogen bonds and Br···O contacts.

## supplementary crystallographic information

### Comment

Three derivatives of 3-oxauracil (4-methyl, 4-bromo, and 4,5 dibromo) were prepared in route to the synthesis of 1-aza -1,3-butadienes. The synthesis of these compounds has previously been reported by Warren *et al.* (1975) and an improved synthesis of the unsubstituted 3-oxauracil was reported by Rehberg & Glass (1995). The synthesis reported herein for the title compound is analogous. The structure of the unsubstituted 3-oxauracil and its monohydrate has been reported by Copley *et. al.* (2005). The hydrogen bonding networks in the three derivatives differ significantly (see also: Parrish, Leuschner *et al.*, 2009; Parrish, Glass *et al.*, 2009).

In the title compound there are two crystallographically independent molecules in the asymmetric unit (Fig. 1). These two molecules are arranged in a planar, pseudo-2-fold screw relationship, as shown in Figure 2. There is a hydrogen bond between the two molecules, N3···O2A, and between the second molecule with a translation related molecule one, N3A···O2C. These two hydrogen bonds are not related by crystallographic symmetry.

There are short, non-bonded contacts between the bromines and the O6 oxygen of the translation related molecules (Fig. 3). A search of the Cambridge Structural Database finds only 10 structures with Br···O=C intermolecular distances of 2.9 Å or less. In the title structure these intermolecular distances are 2.843 (4) Å and 2.852 (4) Å. For example, similar structure, 5-Bromopyrimidin-2(1*H*)-one reported by Yathirajan *et al.* (2007) has a Br···O=C intermolecular distance of 2.895Å [based on coordinates reported in the Cambridge Structural Database (Version 5.30; Allen *et al.*, 2002) as refcode JEVVOW].

### Experimental

Bromomaleic anhydride (3-bromofuran-2,5-dione, 2.0 ml, 22 mmol) was disolved in 10 ml dichloromethane and and trimethylsilyl azide (3.1 ml, 23 mmol) were added dropwise maintaining the reaction temperature below 278K. The solution was stirred under nitrogen for 4 h and then at room temperature for 20 h. To the suspension was added absolute ethanol (6 ml). The resulting mixture was stirred at room temperature for an additional 2 hrs. The white precipitate was filtered, washed with dichlormethane, and then dried *in vacuo* to give the final compound as a white solid (0.85 g, 21%).

### Refinement

Hydrogen positions were calculated and refined using a riding model using the following C—H distances: methylene 0.93 Å, and N—H 0.86 Å. The U_iso_ values for the H atoms were set at 20% above that of the bonded C or N atom.

### Crystal data

**Table:**

C_4_H_2_BrNO_3_	*F*(000) = 736
*M**_r_* = 191.98	*D*_x_ = 2.224 Mg m^−^^3^*D*_m_ = 2.21 Mg m^−^^3^*D*_m_ measured by floatation in Bromoform/Hexane solution
Orthorhombic, *P*22_1_2_1_	Mo *K*α radiation, λ = 0.71073 Å
Hall symbol: P 2bc 2	Cell parameters from 25 reflections
*a* = 7.8913 (12) Å	θ = 10.4–13.1°
*b* = 11.8481 (16) Å	µ = 7.09 mm^−^^1^
*c* = 12.264 (2) Å	*T* = 293 K
*V* = 1146.6 (3) Å^3^	Clear plate, colorless
*Z* = 8	0.45 × 0.20 × 0.10 mm

### Data collection

**Table:**

Siemens R3m/V diffractometer	1975 reflections with *I* > 2σ(*I*)
Radiation source: fine-focus sealed tube	*R*_int_ = 0.0000
graphite	θ_max_ = 27.6°, θ_min_ = 2.4°
θ–2θ scans	*h* = −10→0
Absorption correction: ψ scan (program? reference?)	*k* = −15→0
*T*_min_ = 0.246, *T*_max_ = 0.492	*l* = −15→15
2649 measured reflections	3 standard reflections every 50 reflections
2649 independent reflections	intensity decay: none

### Refinement

**Table:**

Refinement on *F*^2^	Secondary atom site location: difference Fourier map
Least-squares matrix: full	Hydrogen site location: inferred from neighbouring sites
*R*[*F*^2^ > 2σ(*F*^2^)] = 0.038	H-atom parameters constrained
*wR*(*F*^2^) = 0.092	*w* = 1/[σ^2^(*F*_o_^2^) + (0.0593*P*)^2^] where *P* = (*F*_o_^2^ + 2*F*_c_^2^)/3
*S* = 0.95	(Δ/σ)_max_ = 0.001
2649 reflections	Δρ_max_ = 0.85 e Å^−^^3^
163 parameters	Δρ_min_ = −0.53 e Å^−^^3^
0 restraints	Absolute structure: Flack (1983), 1123 Friedel pairs
Primary atom site location: structure-invariant direct methods	Flack parameter: 0.000 (17)

### Special details

**Table:**

Geometry. All e.s.d.'s (except the e.s.d. in the dihedral angle between two l.s. planes) are estimated using the full covariance matrix. The cell e.s.d.'s are taken into account individually in the estimation of e.s.d.'s in distances, angles and torsion angles; correlations between e.s.d.'s in cell parameters are only used when they are defined by crystal symmetry. An approximate (isotropic) treatment of cell e.s.d.'s is used for estimating e.s.d.'s involving l.s. planes.
Refinement. Refinement of *F*^2^ against ALL reflections. The weighted *R*-factor *wR* and goodness of fit *S* are based on *F*^2^, conventional *R*-factors *R* are based on *F*, with *F* set to zero for negative *F*^2^. The threshold expression of *F*^2^ > σ(*F*^2^) is used only for calculating *R*-factors(gt) *etc*. and is not relevant to the choice of reflections for refinement. *R*-factors based on *F*^2^ are statistically about twice as large as those based on *F*, and *R*- factors based on ALL data will be even larger.Successful refinement of the structure in space group P22~1~2~1~ confirms the assignment of this symmetry, which was not the intial choice based on the systematic absences. The pseudo-2-fold screw between the two molecules in the asymmetric unit likely results in the near extinction of the h = 2n + 1 reflections in the h00 line. Only two reflections, -7 0 0 and -9 0 0, have an observed structure factor with a sigma greater than 1, approximately 2. The agreement of the observed and calculated structure factors for these two reflections is good. Although these reflections are, indeed, quite weak the observed structure factors are 2 to 10 times the those of the unobserved k = 2n + 1 and l = 2n + 1 reflections on the 0k0 and 00l lines. These screw-required absent reflections have an intensity of less than one sigma.Note: Checkcif offers conflicting instructions on the choice of the space group. Oiginally solved as P2\~1\~2\~1\~2 checkcif gave PLAT158: Unless for special reasons related to the structure/content, a unitcell and structure is best reported with reference to the Niggli Reduced Cell. Thus I redid the structure as P22\~1\~2\~1\~ and checkcif gave PLAT128 The reported monoclinic space-group is in a non-standard setting. Transformation to the conventional setting is indicated unless there is a good (scientific) reason not to do so.I assume the check for standard reduced cell trumpts the check for non-standard monoclinic space-group setting

### Fractional atomic coordinates and isotropic or equivalent isotropic displacement parameters (Å^2^)

**Table:**

	*x*	*y*	*z*	*U*_iso_*/*U*_eq_	
O1	0.7479 (4)	0.1954 (3)	1.0268 (3)	0.0408 (10)	
C2	0.6216 (7)	0.1447 (5)	0.9687 (5)	0.0426 (14)	
O2	0.6557 (5)	0.0824 (4)	0.8955 (4)	0.0591 (13)	
N3	0.4615 (5)	0.1735 (4)	0.9981 (4)	0.0396 (12)	
H3	0.3776	0.1423	0.9651	0.047*	
Br4	0.19716 (8)	0.27493 (5)	1.10658 (5)	0.04118 (17)	
C4	0.4291 (7)	0.2498 (5)	1.0778 (5)	0.0351 (14)	
C5	0.5485 (7)	0.3026 (5)	1.1320 (6)	0.0417 (16)	
H5	0.5221	0.3540	1.1867	0.050*	
C6	0.7231 (7)	0.2779 (5)	1.1037 (5)	0.0431 (14)	
O6	0.8479 (5)	0.3214 (5)	1.1413 (5)	0.0605 (15)	
O1A	0.2522 (4)	−0.0212 (4)	0.7657 (3)	0.0419 (10)	
C2A	0.1283 (7)	0.0287 (5)	0.8248 (5)	0.0422 (14)	
O2A	0.1627 (5)	0.0880 (4)	0.8988 (4)	0.0570 (13)	
N3A	−0.0342 (6)	0.0020 (4)	0.7945 (4)	0.0399 (12)	
H3A	−0.1178	0.0280	0.8318	0.048*	
Br4A	−0.29870 (8)	−0.08742 (5)	0.67770 (5)	0.04625 (19)	
C4A	−0.0658 (6)	−0.0657 (5)	0.7057 (5)	0.0364 (14)	
C5A	0.0530 (8)	−0.1107 (6)	0.6454 (6)	0.0497 (18)	
H5A	0.0261	−0.1559	0.5858	0.060*	
C6A	0.2254 (8)	−0.0883 (5)	0.6738 (5)	0.0459 (15)	
O6A	0.3514 (5)	−0.1203 (5)	0.6290 (5)	0.0693 (18)	

### Atomic displacement parameters (Å^2^)

**Table:**

	*U*^11^	*U*^22^	*U*^33^	*U*^12^	*U*^13^	*U*^23^
O1	0.0207 (18)	0.052 (3)	0.050 (2)	0.0026 (15)	−0.0004 (16)	−0.004 (2)
C2	0.022 (2)	0.053 (4)	0.052 (4)	−0.001 (3)	0.006 (3)	−0.007 (3)
O2	0.040 (3)	0.075 (3)	0.062 (3)	0.000 (2)	0.005 (2)	−0.039 (3)
N3	0.021 (2)	0.048 (3)	0.049 (3)	−0.004 (2)	0.003 (2)	−0.009 (2)
Br4	0.0184 (2)	0.0541 (3)	0.0510 (3)	0.0028 (3)	0.0026 (3)	−0.0047 (3)
C4	0.025 (3)	0.041 (3)	0.039 (3)	−0.004 (2)	0.004 (2)	−0.001 (3)
C5	0.025 (3)	0.050 (4)	0.050 (4)	0.009 (2)	0.004 (2)	−0.009 (3)
C6	0.020 (3)	0.052 (3)	0.057 (4)	−0.001 (3)	0.004 (3)	−0.006 (3)
O6	0.022 (2)	0.078 (3)	0.081 (4)	−0.002 (2)	−0.001 (2)	−0.033 (3)
O1A	0.0177 (16)	0.054 (2)	0.054 (3)	−0.0022 (15)	−0.0026 (16)	−0.003 (2)
C2A	0.027 (3)	0.053 (4)	0.046 (4)	−0.009 (3)	−0.005 (3)	−0.004 (3)
O2A	0.032 (2)	0.082 (3)	0.057 (3)	−0.008 (2)	−0.005 (2)	−0.022 (3)
N3A	0.023 (2)	0.051 (3)	0.046 (3)	−0.002 (2)	0.000 (2)	−0.010 (2)
Br4A	0.0177 (2)	0.0591 (4)	0.0620 (4)	−0.0024 (3)	−0.0031 (3)	−0.0144 (3)
C4A	0.020 (3)	0.040 (3)	0.049 (4)	−0.003 (2)	−0.005 (2)	0.002 (3)
C5A	0.029 (3)	0.066 (4)	0.054 (4)	0.001 (3)	−0.003 (3)	−0.019 (3)
C6A	0.028 (3)	0.059 (4)	0.052 (4)	0.003 (3)	0.004 (3)	−0.006 (3)
O6A	0.0171 (19)	0.106 (4)	0.084 (4)	0.000 (2)	0.003 (2)	−0.040 (3)

### Geometric parameters (Å, °)

**Table:**

O1—C2	1.365 (7)	O1A—C2A	1.353 (7)
O1—C6	1.372 (7)	O1A—C6A	1.395 (7)
C2—O2	1.193 (7)	C2A—O2A	1.179 (7)
C2—N3	1.358 (7)	C2A—N3A	1.372 (7)
N3—C4	1.355 (8)	N3A—C4A	1.375 (8)
N3—O2A	2.841 (6)	N3A—O2^i^	2.903 (6)
N3—H3	0.8600	N3A—H3A	0.8600
Br4—C4	1.888 (5)	Br4A—C4A	1.887 (5)
Br4—O6^i^	2.843 (4)	Br4A—O6A^i^	2.852 (4)
C4—C5	1.312 (8)	C4A—C5A	1.308 (8)
C5—C6	1.451 (7)	C5A—C6A	1.429 (8)
C5—H5	0.9300	C5A—H5A	0.9300
C6—O6	1.203 (7)	C6A—O6A	1.198 (7)
			
C2—O1—C6	124.7 (4)	O2A—C2A—O1A	120.4 (5)
O2—C2—N3	124.5 (6)	O2A—C2A—N3A	124.1 (6)
O2—C2—O1	120.0 (5)	O1A—C2A—N3A	115.4 (5)
N3—C2—O1	115.4 (5)	C2A—O2A—N3	137.2 (4)
C4—N3—C2	122.4 (5)	C2A—N3A—C4A	121.2 (5)
C4—N3—O2A	113.0 (3)	C2A—N3A—O2^i^	126.6 (4)
C2—N3—O2A	124.6 (4)	C4A—N3A—O2^i^	112.1 (3)
C4—N3—H3	118.8	C2A—N3A—H3A	119.4
C2—N3—H3	118.8	C4A—N3A—H3A	119.4
C4—Br4—O6^i^	177.0 (2)	C4A—Br4A—O6A^i^	178.4 (2)
C5—C4—N3	123.2 (5)	C5A—C4A—N3A	123.8 (5)
C5—C4—Br4	121.8 (5)	C5A—C4A—Br4A	122.7 (5)
N3—C4—Br4	115.0 (4)	N3A—C4A—Br4A	113.6 (4)
C4—C5—C6	117.7 (6)	C4A—C5A—C6A	117.9 (6)
C4—C5—H5	121.1	C4A—C5A—H5A	121.0
C6—C5—H5	121.1	C6A—C5A—H5A	121.0
O6—C6—O1	116.9 (5)	O6A—C6A—O1A	115.2 (5)
O6—C6—C5	126.8 (6)	O6A—C6A—C5A	128.3 (6)
O1—C6—C5	116.3 (5)	O1A—C6A—C5A	116.6 (5)
C2A—O1A—C6A	124.9 (4)		

Symmetry codes: (i) *x*−1, *y*, *z*.

### Hydrogen-bond geometry (Å, °)

**Table:**

*D*—H···*A*	*D*—H	H···*A*	*D*···*A*	*D*—H···*A*
N3—H3···O2A	0.86	1.99	2.841 (6)	171
N3A—H3A···O2^i^	0.86	2.05	2.903 (6)	169

Symmetry codes: (i) *x*−1, *y*, *z*.
